# Structural plasticity: mechanisms and contribution to developmental psychiatric disorders

**DOI:** 10.3389/fnana.2014.00123

**Published:** 2014-11-03

**Authors:** Yann Bernardinelli, Irina Nikonenko, Dominique Muller

**Affiliations:** Department of Basic Neurosciences, University of Geneva Medical SchoolGeneva, Switzerland

**Keywords:** dendritic spines, excitatory synapses, plasticity, morphology, astrocyte

## Abstract

Synaptic plasticity mechanisms are usually discussed in terms of changes in synaptic strength. The capacity of excitatory synapses to rapidly modify the membrane expression of glutamate receptors in an activity-dependent manner plays a critical role in learning and memory processes by re-distributing activity within neuronal networks. Recent work has however also shown that functional plasticity properties are associated with a rewiring of synaptic connections and a selective stabilization of activated synapses. These structural aspects of plasticity have the potential to continuously modify the organization of synaptic networks and thereby introduce specificity in the wiring diagram of cortical circuits. Recent work has started to unravel some of the molecular mechanisms that underlie these properties of structural plasticity, highlighting an important role of signaling pathways that are also major candidates for contributing to developmental psychiatric disorders. We review here some of these recent advances and discuss the hypothesis that alterations of structural plasticity could represent a common mechanism contributing to the cognitive and functional defects observed in diseases such as intellectual disability, autism spectrum disorders and schizophrenia.

## Introduction

Dendritic spines are the major site for excitatory transmission in the brain. They are usually contacted by en passant presynaptic terminals and most often surrounded by astrocytic processes, forming complex structures that dysplay a high degree of functional and structural plasticity. While most research attention has usually focused on the functional aspects of synaptic plasticity and their key contribution to learning and memory mechanisms, work in the last decade has clearly demonstrated the importance of the associated structural rearrangements. These consist of different types of morphological changes (enlargement, growth, pruning, stabilization), affecting different partners (spines, terminals, astrocytic processes) and taking place on different time scales (minutes to days), making them sometimes difficult to relate to the functional changes. These structural rearrangements are also tightly controlled by activity, they are usually NMDA receptor dependent, and have the potential to significantly affect the development and organization of local synaptic networks. Recent advances have started to unravel some of complex molecular mechanisms and signaling systems regulating these synaptic rearrangements, notably at the postsynaptic level. We will therefore mainly focus on these aspects in this review and highlight the multiplicity of mechanisms that may affect structural plasticity and the development of synaptic networks and thereby contribute to cognitive disorders.

## Morphological variability of excitatory synapses

A particular characteristic of dendritic spines is the high variability of their morphological organization. They display major variations in volume, with large spines being several hundred times larger than small spines, but also in length, shape and content in organelles such as ribosomes, endosomal systems or spine apparatus. This high morphological variability is believed to reflect different functional properties of excitatory synapses linked to the size of the postsynaptic density, the number of postsynaptic receptors inserted in the postsynaptic density, the strength of the synapse, its developmental stage or even its stability over time. It is thus often considered that spine morphology correlates with functional parameters (Bourne and Harris, 2008). Large spines usually referred to as mushroom type of spines, or sometimes also as memory spines, are associated with mature, stable synapses that have been strengthened through a process of activity- or plasticity-mediated enlargement. In contrast, thin, elongated spines with small heads, sometimes called learning spines, are interpreted as representing young, newly formed synaptic structures that are more likely to be eliminated over time (Bourne and Harris, 2007). Several *in vitro* and *in vivo* studies have shown a high correlation between the size of the spine head, the size of the postsynaptic density, the size of glutamate-evoked responses and the stability of the spine (Kasai et al., 2003; Matsuzaki et al., 2004). In addition to thin spines, another type of protrusion often analyzed separately are filopodia, usually characterized by the absence of enlargement at the tip. Filopodia are believed to represent precursors of dendritic spines (Ziv and Smith, 1996; Petrak et al., 2005; Toni et al., 2007; Kayser et al., 2008) and they are mainly seen during early stages of development, where they can represent up to 20% of all protrusions. In adolescent and adult tissue however, they constitute only a few percent of all protrusions depending upon criteria used to identify them and their function remains largely unclear as a majority of filopodia just appear and disappear without transforming into spine synapses (Zuo et al., 2005; De Roo et al., 2008a). Overall, based on our current understanding of spine properties, it seems likely that the high morphological variability of dendritic spines reflects the different stages of maturation of excitatory synapses and their individual history. Accordingly, alterations of spine morphology or spine density, as seen in some developmental psychiatric disorders, are often interpreted as indicating defects in spine morphogenesis, stability or plasticity. A more in depth analysis of the dynamic properties of dendritic spines could however provide a better understanding of the underlying defects.

## Mechanism contributing to structural plasticity

A major breakthrough made possible by the development of *in vivo* confocal imaging approaches has been the demonstration that dendritic spines are highly plastic structures that not only continuously change in shape over time (Matus, 2000; Yasumatsu et al., 2008), but can also be formed and eliminated throughout life in an activity-dependent manner (Lendvai et al., 2000; Holtmaat et al., 2005; Caroni et al., 2012). These observations support the concept of a dynamic regulation of excitatory synaptic networks by activity and plasticity mechanisms. A striking aspect of this dynamic regulation of connectivity is its magnitude during periods of intense development such as critical periods and the fact that it then decreases with age (Zuo et al., 2005; Holtmaat and Svoboda, 2009). This is consistent with the notion that the control of spine dynamics by activity plays a central role in shaping the organization of local synaptic networks during development, whereas the reduced structural plasticity still present in older animals maintains possibilities of adaptation while minimizing the possible disruption of existing circuits.

These synaptic rearrangements involve multiple mechanisms to control the formation, maintenance and elimination of excitatory synapses. It is very likely that distinct molecular pathways regulate these different mechanisms independently, as specific manipulations can selectively affect formation, stabilization or elimination of protrusions (Mendez et al., 2010b; Koleske, 2013; Kehoe et al., 2014). Additionally there probably exists homeostatic regulations that coordinate these processes and can compensate for alterations of one or the other mechanism. For example, we have observed that transfection of pyramidal neurons with a dominant-negative mutant of N-cadherin, that strongly reduces spine stability, also leads to a compensatory increase in spine formation (Mendez et al., 2010a). Finally, these different mechanisms are finely tuned by activity and plasticity induction, adding another complexity to their regulation.

**Figure 1:**
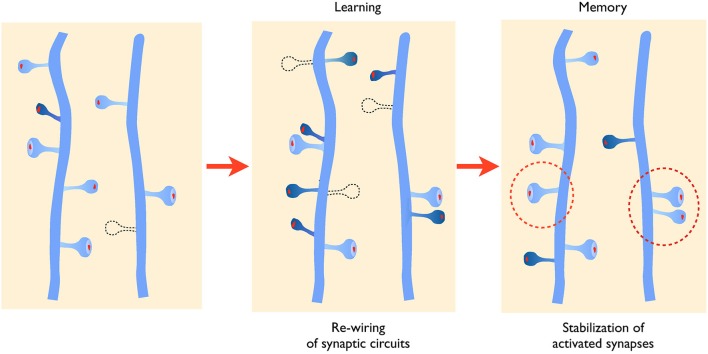
**Activity-mediated structural plasticity**. Left panel: synaptic networks are characterized by a continuous process of growth (dark blue spine) and elimination (dotted line) of dendritic spines that is developmentally regulated. Middle panel: during learning or learning related activity, baseline turnover is significantly enhanced leading to an increased formation and elimination of synaptic contacts, thus allowing remodeling and adaptation of connectivity. New spines also tend to grow in the proximity of activated synapses favoring the formation of spine clusters. Right panel: newly formed and activated synapses are preferentially stabilized (dotted circles) allowing to maintain important functional connections.

In the context of learning related paradigms (LTP), there appear to be two major structural types of changes that have been reported both in *in vitro* systems such as the hippocampal organotypic slice culture or under *in vivo* conditions in anesthetized mice (De Roo et al., 2008b; Hübener and Bonhoeffer, 2010). In slice cultures, induction of long-term potentiation results in two major changes: an increase in spine turnover, characterized by an increase in spine growth but also in spine elimination (Engert and Bonhoeffer, 1999; Nägerl et al., 2004; De Roo et al., 2008b), and a selective stabilization of activated synapses (De Roo et al., 2008b; Hill and Zito, 2013). In living mice, similar changes have also been observed in the visual or motor cortices (Keck et al., 2008; Xu et al., 2009). Notably, in mice trained for a dexterity motor task, spine turnover increases and correlates with the ability to learn the task, while there is also a specific stabilization of newly formed spines that correlates with the memory or retention of the task (Xu et al., 2009; Yang et al., 2009). Based on this type of results, one can thus propose that changes in spine turnover and changes in spine stability represent two key mechanisms associated with memory that could account for some of its antinomic properties: the capacity to learn, which requires adaptation of existing networks, and the capacity to retain information, which requires to maintain important functional circuits (Caroni et al., 2012; see Figure 1).

## Activity-dependent changes in spine morphology and stability

Identification of stimulated synapses within a network has been and still is one of the important limitations for understanding how activity and plasticity regulate synapse properties. This has been overcome in only a few studies using either 2-photon uncaging of glutamate to achieve local stimulation of identified synapses or calcium imaging coupled to electrical stimulation to reveal functional synapses. Although previous electron microscopic (EM) analyses had already suggested that LTP could be associated with morphological changes (Fifková and Anderson, 1981; Geinisman, 1993; Buchs and Muller, 1996), the early, elegant studies by Kasai group (Matsuzaki et al., 2004) were the first to provide a direct demonstration that induction of plasticity was indeed associated with a rapid increase in size of stimulated synapses and an increased sensitivity to glutamate. Other experiments further showed that this enlargement can last several hours (Zito et al., 2009) and that it is correlated with a reorganization of the actin cytoskeleton (Honkura et al., 2008). These observations were thus perfectly in line with accumulating evidence suggesting an implication of the actin cytoskeleton and various actin-regulatory proteins in both LTP maintenance and spine morphology (Fukazawa et al., 2003; Lisman, 2003; Chen et al., 2007; Bramham, 2008). As previously mentioned and revelaed by the studies in hippocampal slice cultures, the structural changes affecting stimulated synapses are also correlated with a major increase in their long-term stability (De Roo et al., 2008b; Hill and Zito, 2013). Very few stimulated synapses disappeared within the days that followed their stimulation, while in contrast non stimulated synapses located on the same dendrite tended to be eliminated at a faster rate. Whether the change in spine stability is directly related to the activity-dependent enlargement remains however unclear. Spine size and spine stability show indeed a high degree of correlation. Repetitive imaging experiments indicate that the size of spine heads continuously fluctuates over a time scale of days (Holtmaat et al., 2005; Yasumatsu et al., 2008). Furthermore, the fact that the activity-dependent spine enlargement seems to be only transient and to reverse within 24 h, while the improved stability can be measured over days suggests that they are regulated by different mechanisms (De Roo et al., 2008b).

**Figure 2:**
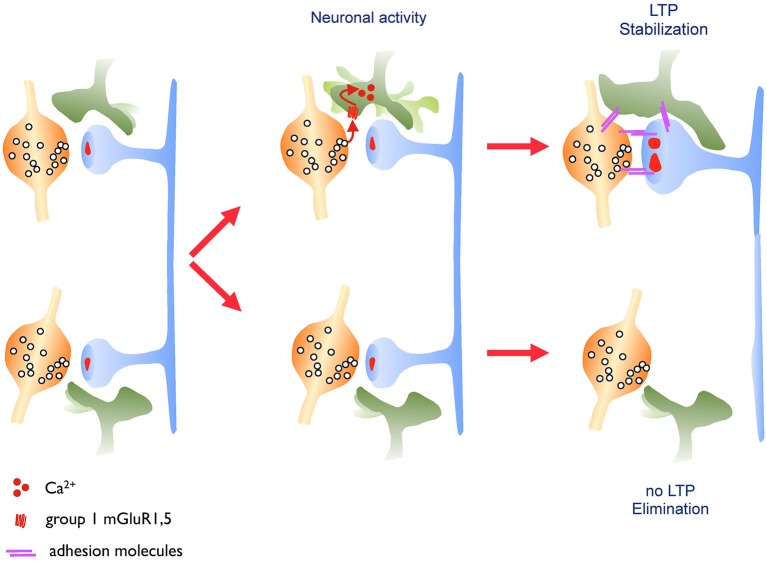
**Astrocytic structural plasticity contributes to the maintenance of activated synapses**. Left panel: excitatory synapses are surrounded by astrocytic processes that show a high level of motility. Middle panel: this astrocytic motility is regulated by the released neurotransmitter, which activates glutamate metabotropic receptors on the astrocytic process leading to an increased calcium flux and motility of the astrocytic process. Right panel: when driven by learning related paradigms (LTP, upper synapse), this enhanced motility leads to an increased and more stable coverage of the synapse by the astrocytic process resulting in a long-lasting stabilization of the synapse. This mechanism might involve, among other possibilities, a contribution of adhesion molecules.

The molecular nature of the mechanisms confering a high stability to activated spines remains also an open issue. Evidence from different studies suggest that the structural organization of the actin cytoskeleton within the spine head could contribute to modify spine stability (Fukazawa et al., 2003; Chen et al., 2007). Phosphorylation of the cytoskeleton-stabilizing protein beta-adducin appears to be required for spine stabilization (Bednarek and Caroni, 2011). Also, several molecules regulating actin polymerization and notably upstream and downstream regulators of Rho GTPases affect the capacity of spines to enlarge and their stability (Rex et al., 2009; Murakoshi et al., 2011; Dubos et al., 2012). Proteins that could also be important to confer stability are adhesion molecules expressed at excitatory synapses. Neuroligins, integrins, ephrins and N-cadherins have all been proposed to contribute to the persistence of spines (Wang et al., 2008; Matter et al., 2009; Bozdagi et al., 2010; McGeachie et al., 2011; Koleske, 2013), although a direct demonstration of their role in long-term activity-dependent spine stability is still often missing. In studies that we carried out to analyze the role of N-cadherin in structural plasticity, we found that an extracellular mutant of N-cadherin strongly affects the life time of dendritic spines and that it prevents activity-mediated spine stabilization (Mendez et al., 2010a). We also showed that N-cadherin is not expressed in all spines and that the spines in which it is expressed show an increased stability. Furthermore N-cadherin expression could be increased through activity patterns that induce LTP, and following stimulation we found that the adhesion molecule selectively accumulates in spines that had been activated. These results thus suggest that modifications in the adhesion profile of activated synapses contribute to regulate the stability and life time of spine synapses (Mendez et al., 2010a). It seems likely that this effect is mediated by increasing trans-synaptic interactions between pre- and postsynaptic structures as molecular complexes showing a periodic organization compatible with the existence of N-cadherin meshwork has been identified in the synaptic cleft of excitatory synapses (Zuber et al., 2005). Adhesion could also be reinforced between synaptic structures and the third partner of the tripartite synapse: the astroglial component. EM studies (Lushnikova et al., 2009) as well as work by Bernardinelli et al. (2014) suggest that synaptic activity and LTP induction can drive a reorganization of astrocytic processes around activated synapses. This glial structural remodeling is mediated by direct activation of glial metabotropic receptors by the released transmitter and calcium influx in the astrocytic process. This transient increase in motility of the astrocytic process leads to a better coverage of the synapse and an enhanced long-term stability of the synaptic contact. This effect has been observed both under *in vitro* and *in vivo* conditions in the somatosensory cortex and is summarized in Figure 2. These new results thus suggest a new role of astrocytic processes in the activity-dependent structural organization of the synapse and a contribution of the astrocyte to the generation of life-long, persistent synapses. In line with these observations, recent work has also demonstrated a critical role of astrocytes in LTP mechanisms and long-term memory in mice (Suzuki et al., 2011). Conversely, there are multiple examples showing that molecular interference with the mechanisms of spine stabilization described above significantly affect cognitive functions (Allen et al., 1998; Sudhof, 2008; Redies et al., 2012; Vukojevic et al., 2012; Ba et al., 2013).

## Mechanisms of activity-dependent spine turnover

The now classical experiments made by Engert and Bonhoeffer (1999) provided the first evidence that induction of plasticity could result in the growth of new dendritic spines. Further studies by several groups confirmed that these new spines display synapses with all the characteristics of morphologically mature and functional contacts (Toni et al., 1999; Nägerl et al., 2007). *In vivo* studies then showed that spine formation and elimination occurred continuously in a developmentally regulated manner and independently of activity (Lendvai et al., 2000; Holtmaat et al., 2005; Zuo et al., 2005). These mechanisms however are strongly enhanced by sensory or motor activity (Holtmaat et al., 2006; Xu et al., 2009). Consistently theta activity or induction of plasticity in hippocampal slices also strongly promotes spine turnover, enhancing both formation and elimination of dendritic spines, leading to a significant activity-dependent rewiring of the hippocampal circuit (De Roo et al., 2008b).

One interesting issue raised by these observations is whether mechanisms of spine growth and elimination are independently regulated. Although activity appears to stimulate both processes simultaneously, several recent results suggest that these two mechanisms are regulated independently of each other. For example, treatment with estrogens results in a significant increase in spine density both in *in vitro* and *in vivo* experiments (Yankova et al., 2001; Sakamoto et al., 2003; Mendez et al., 2010b). Turnover analyses revealed that this effect resulted from a significant increase in spine growth mechanisms, but without changes in spine elimination, thereby accounting for the changes in density. Similarly, specific effects on spine growth mechanisms without alterations of spine elimination have been observed following interference with P21 activated kinase (PAK3; Dubos et al., 2012), by direct activation of the cytoskeletal regulatory protein vasodilator-stimulated phosphoprotein (VASP; Lin et al., 2007; Nikonenko et al., 2013) and following application of BDNF or the phosphatase and tensin homolog (PTEN) inhibitor BpV (Tanaka et al., 2008; Boda et al., 2014). This suggests therefore that mechanisms that promote actin polymerization can directly stimulate spine growth and that signaling pathways implicated in the regulation of protein synthesis such as the PI3K-AKT-mTor pathway participate in the control of this growth process. Conversely, there are also mechanisms that selectively affect spine elimination without altering spine growth processes. This might be the case of long-term depression that has been shown in several recent studies to result in spine shrinkage and disappearance (Becker et al., 2008; Oh et al., 2013; Wiegert and Oertner, 2013). The molecular mechanisms implicated in this effect remain unclear but are likely to require activation of both NMDA and glutamate metabotropic receptors (Oh et al., 2013). Consistent with this interpretation, we recently identified a specific role of the inclusion of the GluN3A subunit in NMDA receptors in the regulation of spine pruning (Kehoe et al., 2013). The GluN3A subunit is highly expressed during critical periods of development and its expression is associated with a decrease in spine density (Roberts et al., 2009). By manipulating the expression of this subunit using gain or loss of function experiments, we found that a central mechanism accounting for these effects is a selective increase in spine pruning associated with expression of the GluN3A subunit (Kehoe et al., 2014). These results thus clearly indicate that spine density can be differentially regulated by interfering with either spine growth or elimination mechanisms.

Whether and how these mechanisms of spine turnover contribute to cognitive functions remains still unclear. However alterations of spine turnover have been reported for cases of intellectual disability or autism spectrum disorders (Cruz-Martín et al., 2010; Pan et al., 2010; Dubos et al., 2012) and mechanisms that regulate spine growth and turnover are also associated with cognitive defects and autism spectrum disorders (Sharma et al., 2010; Cao et al., 2013; Boda et al., 2014).

## Mechanisms underlying spine clustering

**Figure 3:**
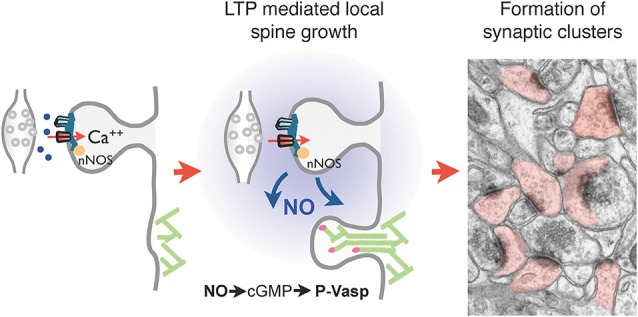
**Regulation of local spine growth by nitric oxide (NO)**. Left panel: neuronal NO synthase (nNOS), present in the postsynaptic density and tightly associated with PSD-95 and NMDA receptors, generates NO upon NMDA receptor activation and calcium influx. Middle panel: NO generated at activated synapses by LTP diffuses locally and triggers the activation of a cGMP-dependent cascade leading to the phosphorylation of the cytoskeleton regulatory protein VASP by cGMP-dependent kinase. This in turn promotes actin filament elongation and spine formation. Right panel: this mechanisms allows the formation of clusters of spine synapses around activated contacts, a phenomenon absent in nNOS deficient mice. The electron microscopy image illustrates clusters of dendritic spines (filled in rose) in the hippocampus of wild type mouse raised in an enriched environment.

An interesting observation related to activity-dependent spine growth mechanisms is that the formation of the new spines occurs in a non-random fashion but is more readily observed in close proximity to activated synapses leading sometimes to the formation of spine clusters (De Roo et al., 2008b; Dubos et al., 2012). This phenomenon, initially detected in hippocampal slice cultures, was then also observed under *in vivo* conditions following a motor learning task (Fu et al., 2012). An intriguing question relates to the mechanism that could promote the growth of new spines in clusters or in regions of activity. Several hypotheses can be considered. First, there is evidence from *in vitro* work that application of glutamate or uncaging of glutamate on a dendrite can rapidly trigger the growth of a new spine (Richards et al., 2005; Kwon and Sabatini, 2011). The phenomenon was shown to depend upon the activity of protein kinases in the postsynaptic dendrite. One could thus consider that intense presynaptic activity and glutamate release could trigger the growth of new spines in small brain areas. One major difficulty with this interpretation is the timing of spine formation. Spine growth was observed to occur within minutes after glutamate uncaging, whereas activity-dependent spine growth is a much slower process that can only be visualized hours after stimulation, long after glutamate release has taken place (Engert and Bonhoeffer, 1999; De Roo et al., 2008b). Although this does not exclude a role for glutamate release, it certainly indicates that activity-induced spine growth is not equivalent to the effects of glutamate uncaging. Another interpretation has recently been proposed based on experiments analyzing the role of nitric oxide (NO) on synapse development (Nikonenko et al., 2013). Nitric oxide can be generated at excitatory synapses in response to NMDA receptor activation of NO synthase, which is closely associated with the postsynaptic density (Burette et al., 2002; Ishii et al., 2006; d’Anglemont de Tassigny et al., 2007). Interventions that blocked NO production or guanilate kinase, the main downstream effector of NO, strongly reduced spine growth mechanisms including the enhancement produced by induction of plasticity (Nikonenko et al., 2013). Conversely, application of cGMP enhanced spine formation, occluding the effects of synaptic activity. Interestingly, these effects could be traced to the phosphorylation of the actin regulatory protein VASP by cGMP kinase. Expression of VASP phospho-mutants that either mimicked or prevented phosphorylation by cGMP kinase reproduced or blocked the effects of activity on spine growth mechanisms (Nikonenko et al., 2013). Moreover in mice deficient in NO synthase, the development of spine synapses was considerably affected and exposure of these mice to an enriched environment failed to promote an increase in synapse density. Analysis of the distribution of spine synapses also revealed a lack of clustering of synapses, a phenomenon readily observed in wild type mice (Nikonenko et al., 2013). These findings thus suggest that one mechanism that may account for the activity-dependent clustering of spines around active sites could involve the production of NO by activated synapses, which in turn stimulates protrusion growth through the phosphorylation of the regulatory protein VASP, promoting in this way the formation of synapses in areas where active partners are found (summary in Figure 3).

While the functional importance of this clustering phenomenon remains still unclear, it is interesting that interference with NO production or signaling is associated with impaired cognitive performance and social dysfunction (Kirchner et al., 2004; Kelley et al., 2009; Tanda et al., 2009; Wass et al., 2009) and variants of the NO synthase 1 gene have been linked to schizophrenia (Shinkai et al., 2002; Bernstein et al., 2011).

## Synaptic defects in developmental psychiatric disorders

All these data highlight the complexity of the mechanisms regulating the dynamics of excitatory synapses during development and strongly suggest that any interference with these structural plasticity properties may have a significant impact on the organization and functional properties of excitatory networks. Changes in spine stability or spine turnover can be expected to affect the number, but also the specificity of excitatory synapses resulting in the formation of synaptic circuits displaying various abnormal features such as hyper- or hypo-connectivity and hyper- or hypo-dynamic responses to activity (Mukai et al., 2008; Pan et al., 2010; Qiu et al., 2011). All these different alterations, by affecting the development and functional properties of synaptic networks, might decrease the reliability of information processing and lead to the formation of maladaptative brain circuits (Sigurdsson et al., 2010). Depending on the molecular mechanism involved, the alterations could affect differentially various brain areas or have a greater impact during different phases of development, thereby accounting for the variability in clinical or behavioral phenotypes. One would also anticipate that alterations of structural plasticity with a strong impact during critical periods of development might have more damaging consequences that could be difficult to reverse and would require very early interventions in order to compensate for the defects. Such early approaches are now tested in many situations related to autism spectrum disorders (Canitano, 2014).

Based on these observations, we propose that a possibly common feature of various developmental psychiatric disorders could be the existence of alterations in the dynamic properties of excitatory synapses. This would be consistent with the strong developmental component of these disorders and the multitude of synaptic proteins possibly implicated. This could also account for the fact that regions showing a high degree of plasticity, such as the prefrontal cortex and the hippocampus, are very often showing functional defects. Finally, in line with this hypothesis, signaling pathways that play an important role in regulating spine dynamics are major candidates for contributing to psychiatric disorders. Examples include Rho GTPases signaling and their regulation of the actin cytoskeleton and the signaling cascades implicated in the control of protein synthesis (Sawicka and Zukin, 2012; Ba et al., 2013). These two signaling systems are particularly important at excitatory synapses to control spine morphology and plasticity, and several genetic alterations associated with psychiatric disorders affect molecules implicated in these two pathways (Boda et al., 2010; Fromer et al., 2014).

The Rho GTPases signaling pathway comprises several molecules that have been associated with intellectual disability, autism spectrum disorders or schizophrenia. Examples include membrane receptors and adhesion molecules able to activate Rho GTPases (cadherins, ephrins), regulators of Rho GTPases (oligophrenin1, GDI1, ARHGEF6, EPAC2, CNK2 (connector enhancer of KSR-2), srGAP3, Disc1, SynGAP), effectors of Rho GTPases (PAK3), as well as cytoskeletal regulatory proteins (Endris et al., 2002; Govek et al., 2004; Nodé-Langlois et al., 2006; Woolfrey et al., 2009; Hayashi-Takagi et al., 2010; Clement et al., 2012; Lim et al., 2014). In many of these cases, alterations of synapse morphology or function have been reported, although analyses of spine dynamics are still often missing. There is one example that we have studied in more details and that concerns the intellectual disability protein PAK3. Several mutations of PAK3 are associated with intellectual disability in humans. Expression of the mutant PAK3 in hippocampal neurons or genetic and pharmacological suppression of PAK3 were all found to result in alterations of spine morphology and spine dynamics (Kreis et al., 2007; Dubos et al., 2012). The two main features observed following interference with PAK3 function were an increase in spontaneous spine growth mechanisms and a block of activity-dependent spine stabilization. Consequently, this resulted in an excessively dynamic synaptic network in which activity failed to selectively stabilize activated synapses (Dubos et al., 2012). This was also reflected by the presence of a high proportion of thin, immature spines and a reduced number of large mushroom type spines (Boda et al., 2004). It can be assumed therefore that these defects resulted in a failure to maintain important connections and to develop specificity in the wiring organization.

The second important pathway strongly implicated in psychiatric disorders involves a number of molecules regulating local protein synthesis. This includes membrane receptors (BDNF and TrkB, Insulin receptor, Ephrins) or adhesion and scaffold molecules (integrins, neuroligin/neurexin complex, shank, SynGAP), intracellular mediators implicated in mTOR signaling (PI3K, Akt, PTEN, TSC2) and regulators of protein synthesis (FMRP, CYFIP1) (Kumar et al., 2005; Lai and Ip, 2009; Mendez et al., 2010a; Sharma et al., 2010; Cuesto et al., 2011; Clement et al., 2012; Boda et al., 2014). This pathway is also critically implicated in the regulation of synaptic strength and in most cases alterations of spine morphology or density have been reported, suggesting impairments of structural plasticity. One of the most studied examples is certainly Fragile X syndrome, associated to multiple CGG repeats in the *fmr1* gene (Bhakar et al., 2012). Mice deficient in FMRP protein show alterations of spine morphology with an increased proportion of thin, elongated spines (Comery et al., 1997, but see also Wijetunge et al., 2014). Recent work using Fmr1 knocout mice provided evidence that these spines are hyper-dynamic, do not respond to sensory stimulation (Pan et al., 2010) and lack stability (Cruz-Martín et al., 2010). In slice culture experiments, we also found that spine turnover was excessively sensitive to activity and more specifically that induction of plasticity failed to induce a differential stabilization of activated spines. Interestingly, in these experiments, we were able to reverse this deficit through application of a PTEN inhibitor, which enhances the PI3K-Akt-mTOR signaling pathway (Boda et al., 2014), while interfering with metabotropic glutamate receptors was only partly effective (Cruz-Martín et al., 2010; Boda et al., 2014). This PTEN inhibitor also improved an associated deficit in LTP mechanisms and when injected in FMR1 deficient mice restored reversal learning in the Morris water maze task (Boda et al., 2014). While much remains to be understood regarding the mechanisms through which local protein synthesis could affect structural plasticity, these data suggest that this system may be an interesting target for modulating properties of spine dynamics and eventually counterbalance some of the deficits observed in the associated disorders.

## Conclusion

Functional synaptic plasticity properties, by quickly changing synaptic strength, allow fast adaptations of network activity which are critical for information processing. However, on a longer time scale, structural plasticity properties may allow a more significant and stable rewiring of synaptic networks through both the formation of new connections and the stabilization of specific contacts. These properties of structural plasticity are particularly important during development where they contribute to shape the structural organization of brain circuits through activity. Molecular analyses of these structural properties started to identify key signaling pathways implicated in these synaptic reorganizations, which also appear to be strong candidates for contributing to cognitive and psychiatric disorders. Hence a common denominator of developmental disorders could involve alterations in spine dynamics that would affect the connectivity and specificity of brain circuits. More systematic analyses of these properties and their functional consequences should allow a better understanding of how they affect information processing and this could eventually lead to new possibilities of treatment of these disorders.

## Conflict of interest statement

The authors declare that the research was conducted in the absence of any commercial or financial relationships that could be construed as a potential conflict of interest.
